# 

**Published:** 1890-06-28

**Authors:** 


### SATURDAY, JUNE 28th, 1890.

**A Tale of Chivalry** . . . . . . . . . 177
**Words of Consolation.**—Prayer . . . . . . 178
**The Doctor's Pulpit.**—The Medical Calf . . . . 179
**The History and Capabilities of Herbal Simples.**—By W. T. Fernie, M.D. XII.—Buckthorn . . . . 180
**Before the Lords' Committee** . . . . . . 181
**Working Women in Large Towns.** V.—Tailoresses 182
**Everybody's Page** . . . . . . . . . 183
**Notes and News** . . . . . . . . . 184
**Editor's Letter-Box.**—The Derby Infirmary . . . 185
**Annotations.**—The Flowers are Calling.—Wanted, A Religion.—Over Specialisation . . . . . . . 186
**The Practitioner's Mirror and Retrospect:**—
Medicine—Recent Views of Enteric Fever . . . . 187
Therapeutics.—Hypnotism . . . . . . . 188
**Turning Over New Leaves.**—On the State of Mind called Attention . . . . . . . . 190
**Mesmerism at the Westminster Aquarium** . . . 191
**Passing Topics.**—Question No. 23 . . . . . 192
**Scraps and Gleanings** . . . . . . . . 192

### EXTRA SUPPLEMENT—THE NURSING MIRROR.

**En Passant.**—The Principle of Co-operation.—Winchester Hospital—Wolverhampton News—Trained Matrons . . xlix

**The Fourth of July** . . . . . . . . . l

**Little Babette** . . . . . . . . . li

**Appointments** . . . . . . . . . lii

**Notes and Queries** . . . . . . . . lii

**Vacancies** . . . . . . . . . . lii

**Amusements and Relaxation** . . . . . . lii

